# Mortality analysis among sepsis patients in and out of intensive care units using the Japanese nationwide medical claims database: a study by the Japan Sepsis Alliance study group

**DOI:** 10.1186/s40560-023-00650-x

**Published:** 2023-01-07

**Authors:** Takehiko Oami, Taro Imaeda, Taka‑aki Nakada, Toshikazu Abe, Nozomi Takahashi, Yasuo Yamao, Satoshi Nakagawa, Hiroshi Ogura, Nobuaki Shime, Yutaka Umemura, Asako Matsushima, Kiyohide Fushimi

**Affiliations:** 1 Department of Emergency and Critical Care Medicine, Chiba University Graduate School of Medicine, 1-8-1 Inohana, Chuo, Chiba, 260-8677 Japan; 2 Health Services Research and Development Center, University of Tsukuba, Tsukuba, Japan; 3 Department of Emergency and Critical Care Medicine, Tsukuba Memorial Hospital, Tsukuba, Japan; 4 Department of Critical Care Medicine, National Center for Child Health and Development, Tokyo, Japan; 5 Department of Traumatology and Acute Critical Medicine, Osaka University Graduate School of Medicine, Osaka, Japan; 6 Department of Emergency and Critical Care Medicine, Graduate School of Biomedical and Health Sciences, Hiroshima University, Hiroshima, Japan; 7 Department of Emergency and Critical Care, Nagoya City University Graduate School of Medical Sciences, Aichi, Japan; 8 Department of Health Policy and Informatics, Tokyo Medical and Dental University Graduate School of Medical and Dental Sciences, Tokyo, Japan

**Keywords:** Sepsis, Diagnosis procedure combination, Intensive care unit, Propensity score matching

## Abstract

**Background:**

A substantial number of sepsis patients require specialized care, including multidisciplinary care, close monitoring, and artificial organ support in the intensive care unit (ICU). However, the efficacy of ICU management on clinical outcomes remains insufficiently researched. Therefore, we tested the hypothesis that ICU admission would increase the survival rate among sepsis patients.

**Methods:**

We conducted a retrospective study using the nationwide medical claims database of sepsis patients in Japan from 2010 to 2017 with propensity score matching to adjust for baseline imbalances. Patients aged over 20 years, with a combined diagnosis of presumed serious infection and organ failure, were included in this study. The primary outcome studied was the in-hospital mortality among non-ICU and ICU patients. In addition to propensity score matching, we performed a multivariable logistic regression analysis for the primary outcome. As the treatment policy was not extracted from the database, we performed sensitivity analyses to determine mortality differences in adults (20 ≤ age ≤ 64), independent patients, patients without malignant tumors, based on the assumption that treatment intensity is likely to increase in those population.

**Results:**

Among 1,167,901 sepsis patients (974,289 in non-ICU and 193,612 in ICU settings), the unadjusted in-hospital mortality was 22.5% among non-ICU patients and 26.2% among ICU patients (3.7% [95% CI 3.5–3.9]). After propensity score matching, the in-hospital mortality was 29.2% among non-ICU patients and 25.8% among ICU patients ($-$ 3.4% [95% CI $-$ 3.7 to $-$ 3.1]). In-hospital mortality with a multivariable regression analysis ($-$ 5.0% [95% CI $-$ 5.2 to $-$ 4.8]) was comparable with the results of the propensity score matching analysis. In the sensitivity analyses, the mortality differences between non-ICU and ICU in adults, independent patients, and patients without malignant tumors were $-$ 2.7% [95% CI $-$ 3.3 to $-$ 2.2], $-$ 5.8% [95% CI $-$ 6.4 to $-$ 5.2], and $-$ 1.3% [95% CI $-$ 1.7 to $-$ 1.0], respectively.

**Conclusions:**

Herein, using the nationwide medical claims database, we demonstrated that ICU admission was potentially associated with decreasing in-hospital mortality among sepsis patients. Further investigations are warranted to validate these results and elucidate the mechanisms favoring ICU management on clinical outcomes.

**Supplementary Information:**

The online version contains supplementary material available at 10.1186/s40560-023-00650-x.

## Background

Sepsis presents dynamic changes in vital signs and life-threatening organ dysfunction through dysregulated inflammation caused by infection [1, 2]. Therefore, a substantial number of sepsis patients require specialized care in the intensive care unit (ICU), including multidisciplinary care, enhanced capacity for monitoring, and multimodal artificial organ support [3, 4]. The concentrated allocation of healthcare supplies in the ICU should be justified based on the assumption that a greater amount of medical resources, including critical care staff, specialized equipment, and medical costs, could improve clinical outcomes in critically ill patients.

While the indication for ICU admission is determined based on the severity of the illness and requirement of life-sustaining interventions [3], some critically ill patients are treated in general wards owing to overcapacity of the ICU, lack of sufficient resources, or treatment policies, such as withholding or withdrawal of intensive therapy [5–7]. A high proportion of septic acute kidney injury patients is treated with continuous renal replacement therapy (RRT) in the ICU [8], whereas a certain number of sepsis patients on mechanical ventilation or vasopressor therapy could be managed in non-ICU settings. Although critically ill patients might have better outcomes if treated in the ICU, no clear recommendations for where sepsis patients should be managed have been stated in the international guidelines [9].

Considering the growing number of patients with sepsis globally [10–14], the potential demand for ICU admission is expected to increase over the next decade. Despite the urgent situation, a large proportion of Japanese patients are on mechanical ventilation in non-ICU settings due to a lower number of ICU beds per person in Japan compared with other developed countries [15–17]. Accordingly, we need to address the impact of ICU admission on clinical consequences for patients with sepsis to develop efficient strategies to deal with the overflow of patients resulting from insufficient ICU services. However, only a few studies have investigated the efficacy of ICU management on clinical outcomes in sepsis patients.

Therefore, we hypothesized that ICU admission increases the survival rate of patients with sepsis. The aim of this study was to investigate the survival rate among sepsis patients in and out of ICU in Japan, where a number of ICU beds per person is comparably lower among developed countries. We conducted a retrospective cohort study using the Japanese nationwide medical claims database from 2010 to 2017.

## Methods

### Study setting and patients

We conducted a retrospective observational study using the Japanese nationwide medical claims database, a Diagnosis Procedure Combination (DPC) system, from 2010 to 2017 [18, 19]. The DPC data were obtained from 1622 hospitals, which covered almost all of acute care facilities, including teaching hospitals or critical care centers authorized ministry of health, in 2017. We selected patients with sepsis along with a combined diagnosis of presumed serious infection and organ dysfunction, as described in a previous report [14]. Patients under 20 years of age were excluded in this study.

The Ethical Review Board of Chiba University Graduate School of Medicine approved this study (approval number: 3429). The review board waived the requirement for written informed consent from the patients or their guardians in accordance with the Ethical Guidelines for Medical and Health Research Involving Human Subjects in Japan.

### Definition of sepsis

Sepsis patients were extracted based on records with presumed serious infection and acute organ dysfunction according to previous literature [11, 14]. Presumed serious infection was defined by the record of antibiotic administration for at least 4 consecutive days. In such cases, antibiotics needed to be administered 48 h before or after the blood culture collection. If death or discharge to other hospitals occurred before the 4 days elapsed, patients with fewer than 4 days of antibiotic administration duration were included in the study. Because laboratory data were unavailable in the database, ICD-10 codes or the records of medical procedures were used for the extraction of organ dysfunction as follows: use of vasopressors, mechanical ventilation or oxygen therapy, initiation of RRT, or diagnostic codes related to kidney dysfunction, hepatic disorder, thrombocytopenia/coagulopathy, or metabolic acidosis (Additional file 1: Table S1). We excluded patients with end-stage renal disease on maintenance dialysis.

### Data extraction and definition

We extracted the following information from the database: age, sex, length of hospital stay, admission to the ICU, primary diagnosis on admission, comorbidities, complications during hospital stay, chronic diseases (malignant tumor, hypertension, diabetes mellitus, heart failure, cerebrovascular disease, ischemic heart disease, chronic respiratory disease, and chronic renal failure), physical function before admission measured by the Barthel Index, site of infection, medical procedures, therapeutic drugs, blood culture tests, medical costs, and number of hospital beds. The average number of hospitalized patients per institution was used as a substitute value indicating the number of hospital beds. Primary diagnosis, comorbidities, and complications were coded based on the International Statistical Classification of Diseases and Related Health Problems 10th revision (ICD-10). In this database, laboratory tests were not available to calculate the Sequential Organ Failure Assessment (SOFA) score. The medical procedures included mechanical ventilation, oxygen therapy, and renal replacement therapy. Therapeutic drugs included vasoactive agents and antibiotics. The site of infection was extracted according to ICD-10 codes as follows: respiratory (mouth, throat, nasal cavity, neck, lung, lower respiratory tract, chest cavity), urogenital (kidney, urinary tract, uterus, genital organs), abdominal (liver, gall bladder, intestine, peritoneal cavity, gastrointestinal system), bone and soft tissue (skin and soft tissue, bone and joint, lymph tissue, breast), meninge/brain/spinal cord, heart, blood, and unknown. Patients with missing data (*n* = 787,261), only regarding the site of infection, were excluded from the analysis. Multiple codes in the “site of infection” were categorized with “Multiple” along with the other infections sites. Community-acquired sepsis was defined as patients whose cultures or antibiotics were initiated within 48 h of hospital admission. Repeat admissions were excluded from the analysis.

The total medical cost per hospitalization was calculated from the fee for drugs, laboratory tests, radiological examinations, and medical procedures during the hospital stay based on reference prices in the Japanese fee schedule, as described in a previous report [20]. The value was adjusted for the admission year according to the consumer price index and converted into U.S. dollars using the latest exchange rate between U.S. dollars and Japanese yen as of February 3rd, 2022 (115.25 yen = $1 USD).

### Statistical analysis

The primary outcome that we studied was the in-hospital mortality among non-ICU and ICU patients. The secondary outcomes were length of hospital stay, ventilator-free days, and total medical cost per hospitalization between the two groups. Because baseline imbalances are most likely observed in non-ICU and ICU patients, we performed propensity score matching analysis. To calculate the propensity scores, we conducted a logistic regression analysis using the demographic variables, comorbidities, and therapies listed in Table 1. Since the indication for RRT in sepsis patients is, in general, determined after ICU admission, we removed RRT from the variable list. Vasopressor administration and respiratory support, including oxygen supplementation and mechanical ventilation, could be initiated in advance of ICU admission. To appropriately adjust severity of illness, we used cardiovascular and respiratory dysfunction at the time of sepsis onset as confounding variables according to the previous reports [21, 22]. Because antibiotics administration 48 h before or after the blood culture collection was defined as a requirement for sepsis patients in our study, we extracted patients with vasopressor therapy, oxygen therapy, or mechanical ventilation within 2 days of the blood culture draw as variables for cardiovascular and respiratory dysfunction. As the incidence of sepsis and the mortality rates in sepsis patients changed year by year [14], we added admission year on the adjustment variables to perform propensity matching. Nearest-neighbor matching was conducted for non-ICU and ICU patients (1:1 matching) according to the propensity scores without replacement. The caliper width was set at 20% of the standard deviation of the propensity scores [23]. To assess the appropriateness of matching, we calculated the absolute standardized mean differences in the covariables and regarded ≤ 10% as a negligible imbalance between the two groups [24]. Following the propensity score matching analysis, we also performed a multivariable logistic regression analysis to adjust the same variables that we used to calculate the propensity scores.

**Table 1:** Clinical characteristics before and after propensity score matching

	Before matching			After matching		
	Non-ICU (*n* = 974,289)	ICU (*n* = 193,612)	SMD	Non-ICU (*n* = 165,344)	ICU (*n* = 165,344)	SMD
Age, year	78 (68–85)	73 (63–81)	0.31	74 (63–82)	74 (64–81)	0.009
Male, *n* (%)	557,742 (57.2)	121,713 (62.9)	0.11	102,969 (62.3)	102,560 (62.0)	0.005
Chronic diseases						
Malignant tumor, *n* (%)	313,354 (32.2)	51,103 (26.4)	0.12	47,119 (28.5)	46,447 (28.1)	0.009
Hypertension, *n* (%)	242,149 (24.9)	48,790 (25.2)	0.01	42,079 (25.4)	42,154 (25.5)	0.001
Diabetes mellitus, *n* (%)	205,238 (21.1)	44,445 (23.0)	0.05	38,128 (23.1)	38,024 (23.0)	0.002
Heart failure, *n* (%)	171,188 (17.6)	46,266 (23.9)	0.16	38,060 (23.0)	37,754 (22.8)	0.004
Stroke, *n* (%)	132,835 (13.6)	30,127 (15.6)	0.05	26,254 (15.9)	25,797 (15.6)	0.008
Ischemic heart disease, *n* (%)	81,159 (8.3)	28,843 (14.9)	0.21	22,825 (13.8)	22,812 (13.8)	0.0002
Chronic respiratory disease, *n* (%)	98,167 (10.1)	13,027 (6.7)	0.12	11,829 (7.2)	11,812 (7.1)	0.0004
Chronic renal failure, *n* (%)	34,043 (3.5)	9932 (5.1)	0.08	8113 (4.9)	8079 (4.9)	0.001
Barthel Index^a^, *n* (%)						
0–60	304,088 (31.2)	47,251 (24.4)	0.15	41,792 (25.3)	41,958 (25.4)	0.002
61–99	104,276 (10.7)	11,909 (6.2)	0.16	10,742 (6.5)	11,003 (6.7)	0.006
100	243,304 (25.0)	47,413 (24.5)	0.01	41,651 (25.2)	41,752 (25.3)	0.001
Community-acquired sepsis, *n* (%)	607,104 (62.3)	82,232 (42.5)	0.41	73,049 (44.2)	74,269 (44.9)	0.01
Infection site						
Abdominal, *n* (%)	130,230 (13.4)	32,261 (16.7)	0.09	27,768 (16.8)	27,459 (16.6)	0.005
Blood, *n* (%)	811 (0.1)	97 (0.1)	0.01	94 (0.1)	93 (0.1)	0.0003
Bone and soft tissue, *n* (%)	35,776 (3.7)	6,343 (3.3)	0.02	5699 (3.4)	5591 (3.4)	0.004
Heart, *n* (%)	4053 (0.4)	4203 (2.2)	0.16	2474 (1.5)	2562 (1.5)	0.004
Meninges/brain/spinal cord, *n* (%)	9963 (1.0)	4223 (2.2)	0.09	3696 (2.2)	3404 (2.1)	0.01
Respiratory, *n* (%)	355,171 (36.5)	52,847 (27.3)	0.20	45,676 (27.6)	46,551 (28.2)	0.003
Urogenital, *n* (%)	68,981 (7.1)	8752 (4.5)	0.11	8195 (5.0)	8195 (5.0)	0.01
Multiple, *n *(%)	274,869 (28.2)	58,962 (30.5)	0.05	50,516 (30.6)	50,193 (30.4)	0.004
Unknown, *n* (%)	94,435 (9.7)	25,924 (13.4)	0.11	21,226 (12.8)	21,296 (12.9)	0.001
Vasopressor therapy, *n* (%)	94,823 (9.7)	54,529 (28.2)	0.48	33,357 (20.2)	40,457 (24.5)	0.10
Vasopressor therapy at the time of sepsis onset, *n* (%)	218,928 (6.6)	50,686 (21.0)	0.42	27,278 (16.5)	28,132 (17.0)	0.01
Oxygen therapy, *n* (%)	804,712 (82.6)	169,436 (87.5)	0.14	127,632 (77.2)	147,090 (89.0)	0.31
Oxygen therapy at the time of sepsis onset, *n* (%)	543,503 (55.8)	77,219 (39.9)	0.32	69,738(42.2)	69,972 (42.3)	0.003
Mechanical ventilation, *n* (%)	103,265 (10.6)	114,689 (59.2)	1.19	53,504 (32.4)	87,179 (52.4)	0.42
Mechanical ventilation at the time of sepsis onset, *n* (%)	56,919 (5.8)	69,375 (35.8)	0.79	41,940 (25.4)	43,647 (26.4)	0.02
Kidney dysfunction, *n* (%)	383,863 (39.4)	121,605 (62.8)	0.48	97,530 (59.0)	97,209 (58.8)	0.004
RRT, *n* (%)	37,653 (3.9)	44,553 (23.0)	0.58	17,403 (10.5)	33,026 (20.0)	0.27
Thrombocytopenia/coagulopathy, *n* (%)	105,106 (10.8)	39,705 (20.5)	0.27	30,141 (18.2)	30,171 (18.2)	0.0005
Hepatic disorder, *n* (%)	39,380 (4.0)	5882 (3.0)	0.05	4918 (3.0)	5071 (3.1)	0.005
Acidosis, *n* (%)	6933 (0.7)	2794 (1.4)	0.07	2039 (1.2)	2057 (1.2)	0.001
Total number of hospital beds, *n* (%)						
≤ 272 beds	274,870 (28.2)	24,159 (12.5)	0.40	23,587 (14.3)	23,232 (14.1)	0.006
273–404 beds	251,498 (25.8)	42,156 (21.8)	0.10	37,657 (22.8)	37,898 (22.9)	0.003
405–587 beds	234,881 (24.1)	54,139 (28.0)	0.09	45,584 (27.6)	46,051 (27.9)	0.006
≥ 588 beds	213,040 (21.9)	73,158 (37.8)	0.35	58,516 (35.4)	58,163 (35.2)	0.005

Data are presented as mean (SD) or median (quartile)

ICU: intensive care unit; SD: standard deviation; SMD: standardized mean difference; RRT: renal replacement therapy

^a^Number of patients missing Barthel index: non-ICU 322,621 and ICU 87,039 (before matching), non-ICU 71,159 and ICU 70,631 (after matching)

As treatment policies, such as withholding or withdrawal of life-sustaining interventions, could not be collected using the database, they could serve as confounding factors for the analysis, despite propensity score matching. Assuming that the intensity of treatment is likely to decrease in older adults, patients with low physical function, and patients with malignant tumors due to the treatment policy [5, 6, 25], we conducted sensitivity analyses according to the age of enrolled patients, physical function, and the prevalence of malignancy. As described in a previous publication [14], we defined adults as 20 ≤ age ≤ 64. In this study, we were unable to determine who were initially admitted to non-ICU but transferred to ICU after that or patients who were transferred from another hospital. As the transferred patients could potentially cause a bias in the clinical outcomes, we performed a sensitivity analysis excluding patients who were transferred to other hospitals. As we removed RRT from the adjustment variables to perform propensity matching, we conducted an analysis without patients on RRT. Along with those variables, we performed sensitivity analyses with regard to vasopressor use, mechanical ventilation, and combination of the interventions.

Continuous variables were expressed as means (standard deviation) or medians (quartiles) and analyzed using the Student’s *t* test or Mann–Whitney *U* test, as deemed appropriate. Categorical variables were presented as numbers and percentages and were examined using Pearson’s chi-square test. The Kaplan–Meier method was used to analyze survival differences between the two groups. We used the Cox regression analysis to estimate the effect of the variable on survival. Statistical significance was determined if the two-tailed *p* value was < 0.05. We conducted data manipulation and statistical analysis using SQL (mariadb v10.4.17), R version 4.1.2 (R Foundation for Statistical Computing, Vienna, Austria, http://www.R-roject.org/), and pandas (v1.0.5), scipy (v1.7.3), numpy (v1.21.4), seaborn (v0.11.2), matplotlib (v3.5.1), and statsmodels (v0.13.2) in Python (v3.9.0).

## Results

### Clinical characteristics in the cohort

Among the 1,167,901 patients with sepsis enrolled in this study, the numbers of non-ICU and ICU patients were 974,289 and 193,612, respectively (Additional file 2: Fig. S1). The median age was 78 (68–85) among the non-ICU patients and 73 (63–81) among the ICU patients. The proportion of men was higher in ICU (62.9%) patients than in non-ICU patients (57.2%). In terms of chronic diseases, the proportion of heart failure and ischemic heart disease was higher among ICU patients, whereas the proportion of malignant tumor and chronic respiratory disease was higher among non-ICU patients. The proportion of infection sites was comparable between the two groups, except for that of respiratory infection. In terms of artificial organ support, the proportions of vasopressor therapy, mechanical ventilation, and RRT among non-ICU and ICU patients were 9.7% and 28.2%, 10.6% and 59.2%, and 3.9% and 23.0%, respectively (Table 1). The number of patients on vasopressor, ventilator, and RRT increased year by year (Additional file 3: Fig. S2). The unadjusted in-hospital mortality was 22.5% among non-ICU patients and 26.2% among ICU patients (3.7% [95% CI 3.5–3.9]) (Table 2).

**Table 2:** Clinical outcomes before and after propensity score matching

	Before matching			After matching		
	Non-ICU (*n* = 974,289)	ICU (*n* = 193,612)	Difference (95% CI)	Non-ICU (*n* = 165,344)	ICU (*n* = 165,344)	Difference (95% CI)
In-hospital mortality, *n* (%)	218,928 (22.5)	50,686 (26.2)	3.7 (3.5 to 3.9)	48,328 (29.2)	42,4701 (25.8)	$$-$$ 3.4 ($$-$$ 3.7 to $$-$$ 3.1)
Length of hospitalization (day)			14.1 (13.6 to 14.6)			2.9 (2.2 to 3.6)
Mean (SD)	42.2 (100.8)	56.3 (66.7)		52.8 (129.3)	55.7 (67.0)	
Median (IQR)	26 (14–50)	40 (21–70)		34 (17–64)	39 (21–69)	
Length of ICU stay (day)			NA			NA
Mean (SD)	NA	7.0 (6.0)		NA	6.4 (5.6)	
Median (IQR)	NA	5 (2–12)		NA	5 (2–10)	
Ventilator-free days			$$-$$ 4.2 ($$-$$ 4.3 to $$-$$ 4.2)			$$-$$ 1.2 ($$-$$ 1.3 to $$-$$ 1.2)
Mean (SD)	25.3 (7.9)	21.0 (10.1)		23.0 (9.6)	21.7 (9.9)	
Median (IQR)	28 (28–28)	27 (18–28)		28 (24–28)	28 (20–28)	
Total medical cost per hospitalization ($)			21,600 (21,500 to 21,700)			11,520 (11,300 to 11,700)
Mean (SD)	16,891 (24,014)	38,489 (39,117)		24,910 (29,982)	36,426 (36,0736)	
Median (IQR)	10,513 (5741–19,913)	27,831 (15,808–48,673)		16,356 (8,685–30,122)	26,144 (14,867–16,040)	

Data are presented as mean (SD) or median (quartile)

ICU: intensive care unit; CI: confidence interval; SD: standard deviation; IQR: interquartile range; NA: not applicable

### Clinical outcomes after propensity score matching

**Fig. 1:**
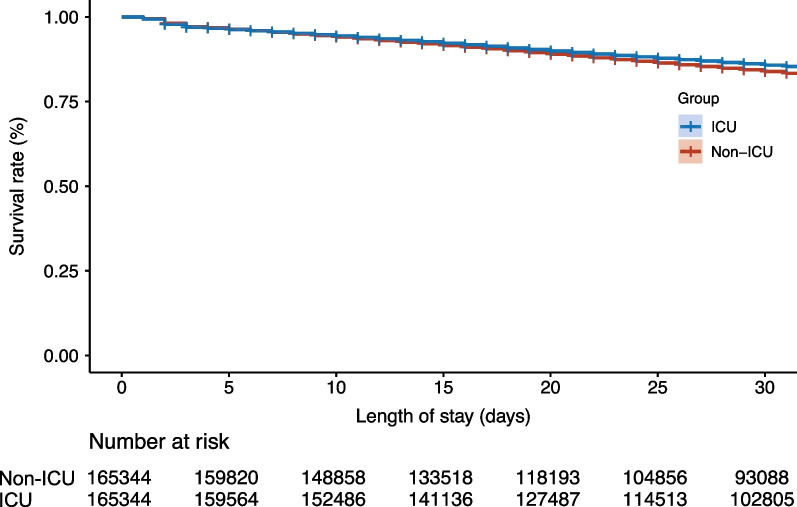
Kaplan–Meier curve for 30-day mortality between non-ICU and ICU patients after propensity score matching. The mortality of intensive care unit (ICU) patients 30 days after admission was significantly lower than that of non-ICU patients after propensity score matching (hazard ratio 0.98 [95% confidence interval 0.97 to 0.99]). Discharged patients were excluded from the analysis

After propensity score matching, the number of non-ICU and ICU patients was 165,344 in both groups (Table 1, Additional file 4: Fig. S3). Patient background was comparable between the two groups except for RRT, with ≤ 10% absolute standardized mean differences in the covariables. The proportions of vasopressor therapy, oxygen therapy, and mechanical ventilation at the time of sepsis onset among non-ICU and ICU patients were 16.5% and 17.0%, 42.2% and 42.3%, and 25.4% and 26.4%, respectively. The length of hospitalization and ventilator-free days in the non-ICU and ICU patients were 34 (17–64) days and 39 (21–69) days, 28 (24–28) and 28 (20–28), respectively. Although the total medical cost per hospitalization was higher among ICU patients ($26,144 [14,867–16,040]) than that among non-ICU patients ($15,269 [7999–28,697]), the difference between the two groups was smaller in the cohort after matching than that before matching. In-hospital mortality was 29.2% and 25.8% among non-ICU and ICU patients, respectively ($$-$$ 3.4% [95% CI $$-$$ 3.7 to $$-$$ 3.1]) (Table 2). The Kaplan–Meier curve also demonstrated a significantly lower mortality at 30 days after hospital admission in ICU patients (hazard ratio 0.98 [95% CI 0.97–0.99]) (Fig. 1).

### Sensitivity analysis

In the subcohorts, which included adults, independent patients, and patients without malignant tumors, the difference in adjusted in-hospital mortality after propensity score matching was $-$ 2.7% [95% CI $-$ 3.3 to $-$ 2.2], $-$ 5.8% [95% CI $-$ 6.4 to $-$ 5.2], and $-$ 1.3% [95% CI $-$ 1.7 to $-$ 1.0], respectively. After excluding patients who were transferred to other hospitals, the in-hospital mortality difference was $-$ 1.5% [95% CI $-$ 1.8 to $-$ 1.1]. After exclusion of patients on RRT, the mortality difference was $-$ 6.2% [95% CI $-$ 6.5 to $-$ 5.9]. The mortality difference in patients on vasopressor, ventilators, or combination of the interventions was greater than that in the overall cohort. In addition, the difference in adjusted in-hospital mortality in the overall cohort with multivariable regression analysis was $-$ 5.0% [95% CI $-$ 5.2 to $-$ 4.8], which was consistent with the results of propensity score matching (Table 3).

**Table 3:** Sensitivity analyses for in-hospital mortality between non-ICU and ICU patients

	Number of patients		Difference (95% CI)
	Non-ICU	ICU	
Overall cohort			
Propensity score matching	165,344	165,344	$-$ 3.4 ($-$ 3.7 to $-$ 3.1)
Multivariable logistic regression	974,289	193,612	$-$ 5.0 ($-$ 5.2 to $-$ 4.8)
Subcohort1: adults (20 ≤ age ≤ 64)			
Propensity score matching	42,628	42,628	$-$ 2.7 ($-$ 3.3 to $-$ 2.2)
Multivariable logistic regression	181,607	53,195	$-$ 2.6 ($-$ 3.0 to $-$ 2.3)
Subcohort2: independent patients (Barthel index 100)			
Propensity score matching	40,488	40,488	$-$ 5.8 ($-$ 6.4 to $-$ 5.2)
Multivariable logistic regression	243,304	47,413	$-$ 5.7 ($-$ 6.1 to $-$ 5.4)
Subcohort3: excluding patients with malignant tumor			
Propensity score matching	118,873	118,873	$-$ 1.3 ($-$ 1.7 to $-$ 1.0)
Multivariable logistic regression	660,935	142,509	$-$ 2.6 ($-$ 2.8 to $-$ 2.3)
Subcohort4: excluding patients who were transferred to other hospitals			
Propensity score matching	118,680	118,680	$-$ 1.5 ($-$ 1.8 to $-$ 1.1)
Multivariable logistic regression	798,575	136,042	$-$ 3.2 ($-$ 3.5 to $-$ 3.0)
Subcohort5: excluding patients on RRT			
Propensity score matching	135,069	135,069	$-$ 6.2 ($-$ 6.5 to $-$ 5.9)
Multivariable regression analysis	936,636	149,059	$-$ 7.7 ($-$ 8.0 to $-$ 7.5)
Subcohort6: patients on vasopressor			
Propensity score matching	38,585	38,585	$-$ 2.8 ($-$ 3.5 to $-$ 2.1)
Multivariable logistic regression	94,823	54,529	$-$ 3.7 ($-$ 4.2 to $-$ 3.1)
Subcohort7: patients on ventilators			
Propensity score matching	73,123	73,123	$-$ 11.1 ($-$ 11.6 to $-$ 10.6)
Multivariable logistic regression	114,689	103,265	$-$ 12.1 ($-$ 12.5 to $-$ 11.7)
Subcohort8: patients on vasopressor and ventilators			
Propensity score matching	11,958	11,958	$-$ 8.4 ($-$ 9.5 to $-$ 7.3)
Multivariable logistic regression	65,666	12,133	$-$ 8.6 ($-$ 9.4 to $-$ 7.8)

ICU: intensive care unit; CI: confidence interval; RRT: renal replacement therapy

## Discussion

In this study, we demonstrated that sepsis patients treated in the ICU exhibited decreased in-hospital mortality compared with those out of the ICU using a propensity score matching analysis. Lower mortality among sepsis patients admitted to the ICU was also presented in the sensitivity analyses and other confounding adjustment analyses, suggesting that the results were robust, regardless of differences in patient backgrounds and treatment intensity.

The advantages of ICU admission over hospitalization in general wards for critically ill patients were consistent with previous reports [17, 26–30]. In a comparative observational study, mechanically ventilated patients hospitalized in the ICU exhibited a higher in-hospital survival rate than those in medical wards with fewer endotracheal tube-related complications [29]. Another study comparing critically ill patients on ventilator support treated in the ICU and high-dependency care units demonstrated decreased in-hospital mortality in the ICU [30]. Although a superior survival rate attributable to ICU management has been demonstrated in critically ill patients, few studies have focused on sepsis patients with regard to the efficacy of ICU management on clinical outcomes. To the best of our knowledge, this study is the first to demonstrate the advantages of ICU admission in patients with sepsis over non-ICU management using confounding adjustment analyses. Although we performed a propensity score matching analysis to adjust for baseline imbalances, there might be other confounding factors that could affect the results. A potential confounder could be the treatment policy, such as withholding or withdrawing from intensive therapies. In the sensitivity analyses concerning age, physical function, and malignancy, the significant advantage in ICU settings over general wards was consistent among all subcohorts. The sensitivity analysis in adults strengthened the robustness that ICU treatment contributed to decreasing mortality. Likewise, the consistency of the decreased mortality among independent and non-malignancy patients admitted to the ICU supports the plausibility of our hypothesis. Future studies should address detailed information about treatment policies in the database.

The exact mechanisms of the advantages in ICU management can be attributed to several factors, including sufficient medical resources and artificial organ support [3, 4]. Mechanical ventilation could be performed in general wards; however, close monitoring might be difficult owing to the lack of adequate resources, leading to mechanical complications, such as accidental extubation and delayed recognition of equipment failures [29]. These errors may worsen clinical outcomes in critically ill patients. In the present study, the differences in mortality between the two groups were greater among patients on mechanical ventilation than among overall cohort, suggesting that sepsis patients on therapeutic interventions have optimal indications for ICU management. Other than artificial organ support, adequacy of antimicrobials, time to antimicrobial initiation, initial fluid resuscitation, and time to shock withdrawal potentially contributed to improving the clinical outcomes in sepsis patients treated in the ICU [31–34]. Future studies are expected to reveal the contribution of those factors on outcomes in sepsis patients managed in the ICU.

Not all sepsis patients are not managed in the ICU due to treatment policies, severity of illness, or lack of medical resources and staffs. Considering that some sepsis patients who were treated in general wards showed favorable outcomes, a certain number of sepsis patients without life-sustaining interventions could be managed in non-ICU settings. As delayed ICU admission was associated with increased in-hospital mortality in the study of 12,380 ward patients [35], timely admission to ICU should be delivered according to physiological deterioration and indication for organ support. Taken together, while sepsis patients without severe organ injuries are possibly treated outside the ICU, those patients need to be cautiously monitored with early warning signs and applicable scoring systems [9, 36, 37].

To provide an appropriate environment where mechanical organ support is performed without iatrogenic complications, consistent ICU services by intensivists and sufficient nurse staffing are warranted. Regarding their optimal allocation, the lack of intensivists in the ICU or lower patient-to-intensivist ratios reportedly increase the mortality of critically ill patients [38, 39]. Although a high-intensity ICU model or closed-ICU, where intensivists are responsible for day-to-day management, is recommended, the benefit of a 24-h service of intensivists remains controversial [3]. Furthermore, nurse staffing also contributes to altering patient outcomes [40, 41]. In Japan, the nurse-to-patient ratio in general wards is 1:7 or higher, whereas the ICU allocates one nurse to two patients. While an appropriate nurse-to-patient ratio is lacking owing to scarce evidence, inadequate nursing staffing increases the in-hospital risk of death through insufficient delivery of basic care [42]. Accordingly, ICU settings with a sufficient number of intensivists and nurses for patients would be preferable for sepsis management, particularly for patients receiving mechanical organ support. In this study, quality indicators such as the availability and number of ICU physicians, nurse–patient ratios, presence or absence of resident clinical engineers and pharmacists, and availability and number of advanced medical equipment were unavailable. Therefore, we used the number of hospital beds implicating the quality of the institution for confounding adjustment [43–45]. Further investigations need to address quality indicators of hospitals to clarify the efficiency of ICU management.

While the abundance of staffing and medical resources in the ICU depends on governmental policies and medical systems in different countries, the number of ICU beds per capita by country also varies widely. Compared with other developed countries, Japan has fewer ICU beds (five beds per 100,000 people). In western countries, the number of ICU beds varies: 3.5 beds per 100,000 population in the U.K., 9.3 beds per 100,000 population in France, 13.5 beds per 100,000 population in Canada, and 20 beds per 100,000 population in the U.S. [16, 46, 47]. In addition to variations in the number of ICU beds, the indications for ICU admission and critical care services vary among these countries. In a demographic study comparing critical care delivery between Japan and the U.S., the details of ICU utilization differed by age population, proportion of postoperative ICU admissions, and severity of the critical illness. In terms of severity, the mean APACHE III score among Japanese patients was higher than that among American patients. These differences might be attributable to medical policies, demographic characteristics, and cultural norms [15]. In this context, our results should be interpreted cautiously in accordance with the characteristics of the healthcare system.

This study, however, has several limitations. First, the medical claims database lacks laboratory data. As a calculation of severity scores, such as the SOFA score, was unavailable, we used organ dysfunctions and therapeutic interventions to adjust imbalances. Although we followed the validated methods using ICD-10 codes for organ dysfunction [21], the results should be interpreted with caution due to the possibility of remained confounding factors. Second, confounding by indication for ICU admission was not adjusted. Discrete decisions by responsible physicians potentially cause biased perceptions of disease severity and prognosis among medical personnel. Third, long-term outcomes were not assessed in this study. We should investigate a long-term effect of ICU management on physical disabilities and cognitive impairment in future research. Fourth, the primary diagnosis for hospitalization in some patients was not sepsis, which indicates that the cause of death might not be related to sepsis. Fifth, treatment policies, such as withholding or withdrawal of life-sustaining interventions, were not recorded due to the study design, which could have affected the mortality of non-ICU patients. As a result, we performed sensitivity analyses by age, physical function, and malignancy to scrutinize the results among populations who are unlikely to be withheld or withdrawn from intensive care. Sixth, this study did not distinguish between high intensity (closed) ICUs and low intensity (open) ICUs in the mortality analysis. Future investigations are warranted to collect detailed information, including treatment policy and differences in ICU management systems, and to elucidate the mechanisms that favor ICU admission for clinical outcomes.

## Conclusions

In this study, using the nationwide medical claims database, we demonstrated that ICU admission was potentially associated with decreased in-hospital mortality among patients with sepsis. Further investigations are still needed to validate these results and to elucidate the mechanistic impact of ICU management.

## Supplementary Information

**Additional file 1: Table S1.** Diagnostic categories with corresponding ICD-10.

**Additional file 2: Figure S1.** Flowchart of study population.

**Additional file 3: Figure S2.** Temporal changes in the number and proportion of sepsis patients on therapeutic interventions in the ICU between 2010 and 2017.

**Additional file 4: Figure S3.** Distribution of propensity score matching.

## Data Availability

The data sets used and analyzed in our study are available from the corresponding author upon reasonable request.

## Abbreviations

ICU: Intensive care unit
DPC: Diagnosis procedure combination
ICD-10: International Statistical Classification of Diseases and Related Health Problems 10th revision
SOFA: Sequential Organ Failure Assessment
RRT: Renal replacement therapy
CI: Confidence interval
