# Supplementary material for: Mortality analysis among sepsis patients in and out of intensive care units using the Japanese nationwide medical claims database: a study by the Japan Sepsis Alliance study group
Source: J Intensive Care. 2023 Jan 7;11:2. doi: 10.1186/s40560-023-00650-x (PMC9826578; doi:10.1186/s40560-023-00650-x)
Supplement: Supplementary file 1 — Additional file 1: Table S1. Diagnostic categories with corresponding ICD-10. [file 40560_2023_650_MOESM1_ESM.docx]

**Mortality analysis among sepsis patients in and out of intensive care units using the Japanese nationwide medical claims database-a study by the Japan Sepsis Alliance study group**

**Authors:** Takehiko Oami^1^, Taro Imaeda^1^, Taka‑aki Nakada^1^, Toshikazu Abe^2,3^, Nozomi Takahashi^1^, Yasuo Yamao^1^, Satoshi Nakagawa^4^, Hiroshi Ogura^5^, Nobuaki Shime^6^, Yutaka Umemura^5^, Asako Matsushima^7^ and Kiyohide Fushimi^8^

**Affiliations:**

1. Department of Emergency and Critical Care Medicine, Chiba University Graduate School of Medicine, Chiba, Japan
2. Health Services Research and Development Center, University of Tsukuba, Tsukuba, Japan
3. Department of Emergency and Critical Care Medicine, Tsukuba Memorial Hospital, Tsukuba, Japan
4. Department of Critical Care Medicine, National Center for Child Health and Development, Tokyo, Japan
5. Department of Traumatology and Acute Critical Medicine, Osaka University Graduate School of Medicine, Osaka, Japan
6. Department of Emergency and Critical Care Medicine, Graduate School of Biomedical and Health Sciences, Hiroshima University, Hiroshima, Japan
7. Department of Emergency & Critical Care, Graduate School of Medical Sciences, Nagoya City University, Aichi, Japan
8. Department of Health Policy and Informatics, Tokyo Medical and Dental University Graduate School of Medical and Dental Sciences, Tokyo, Japan

**Correspondence:**

Taka-aki Nakada

Chiba University Graduate School of Medicine, Department of Emergency and Critical Care Medicine, 1-8-1 Inohana, Chuo, Chiba 260-8677, Japan

Phone: +81-43-226-2372, Fax: +81-43-226-2371,

Email: taka.nakada@nifty.com**Additional files**

**Additional File 1: Table S1.** Diagnostic categories with corresponding ICD-10

**Additional File 2: Figure S1.** Flowchart of study population

**Additional File 3: Figure S2.** Temporal changes in the number and proportion of sepsis patients on therapeutic interventions in the ICU between 2010 and 2017

**Additional File 4: Figure S3.** Distribution of propensity score matching

**Table S1** Diagnostic categories with corresponding ICD-10

| Diagnosis | ICD-10 codes |
| --- | --- |
| Comorbidity |  |
| Malignant tumor | C00-C97, D00-D09 |
| Hypertension | I10-I15 |
| Diabetes mellitus | E10-E14 |
| Heart failure | I50 |
| Ischemic heart disease | I60-I69 |
| Cerebrovascular disease | I20-I25 |
| Chronic respiratory disease | J40-J47 |
| Chronic renal failure | N18 |
| Focus of infection |  |
| Respiratory | A15-A16, J00-J06, J09-J18, J20-J22, J31-J32, J35-J37, J39.0, J39.1, J85-J86 |
| Urogenital | A18.1, A51.0, A54.0-A54.2, A56.0-A56.2, A59.0, A60.0, N30.0, N30.8, N39.0, N41.0-N41.3, N45, N49.0-N49.2, N70-N77, O23 |
| Abdominal | A00-A09, A18.3, A42.1, A74.8, K35-K38, K57.0, K57.2, K57.4, K57.8, K61, K63.0, K63.1, K65, K67, K75.0, K80.0, K80.1, K80.3, K80.4, K81, K83.0 |
| Bone and soft tissue | A18.0, A18.4, A26.0, A28.1, A31.1, A31.8, A32.0, A36.3, A42.2, A43.1, A46, A48.0, L00-L08, M00, M01.0, M46.3, M46.5, M49.1-M49.3, M60.0, M86.0, M86.1, M86.65, M86.66, M86.69, M86.99 |
| Blood | A19, A40.0, A49.0, A49.1, A49.9 |
| Organ dysfunction |  |
| Renal | N00.9, N10, N17.0, N17.1, N17.8, N17.9 |
| Hepatic | K72.0, K72.9, K76.8 |
| Thrombocytopenia | D69.5, D69.6 |
| Coagulopathy | D65, D68.9 |
| Acidosis | E87.2 |

ICD-10, International Statistical Classification of Diseases and Related Health Problems 10th revision.

**Figure legends**

**Figure S1. Flow chart of study population**

**Figure S2. Temporal changes in the number and proportion of sepsis patients on therapeutic interventions in the ICU between 2010 and 2017**

The bar plot depicts the relationship between the year of hospital admission on the x-axis and the number of sepsis patients on (A) vasopressor, (B) ventilator, and (C) renal replacement therapy (RRT) in the ICU per 1,000 patients on the y-axis. The line plot depicts another relationship between the admission year and the proportion of sepsis patients on (A) vasopressor, (B) ventilator, and (C) RRT in the ICU on the y-axis.

**Figure S3. Distribution of propensity score matching**

A: Distribution of propensity score in non-ICU and ICU patients

B: Distribution of propensity score in non-ICU and ICU patients after matching
