# Supplementary figures and images for: Mortality analysis among sepsis patients in and out of intensive care units using the Japanese nationwide medical claims database: a study by the Japan Sepsis Alliance study group
Source: J Intensive Care. 2023 Jan 7;11:2. doi: 10.1186/s40560-023-00650-x (PMC9826578; doi:10.1186/s40560-023-00650-x)

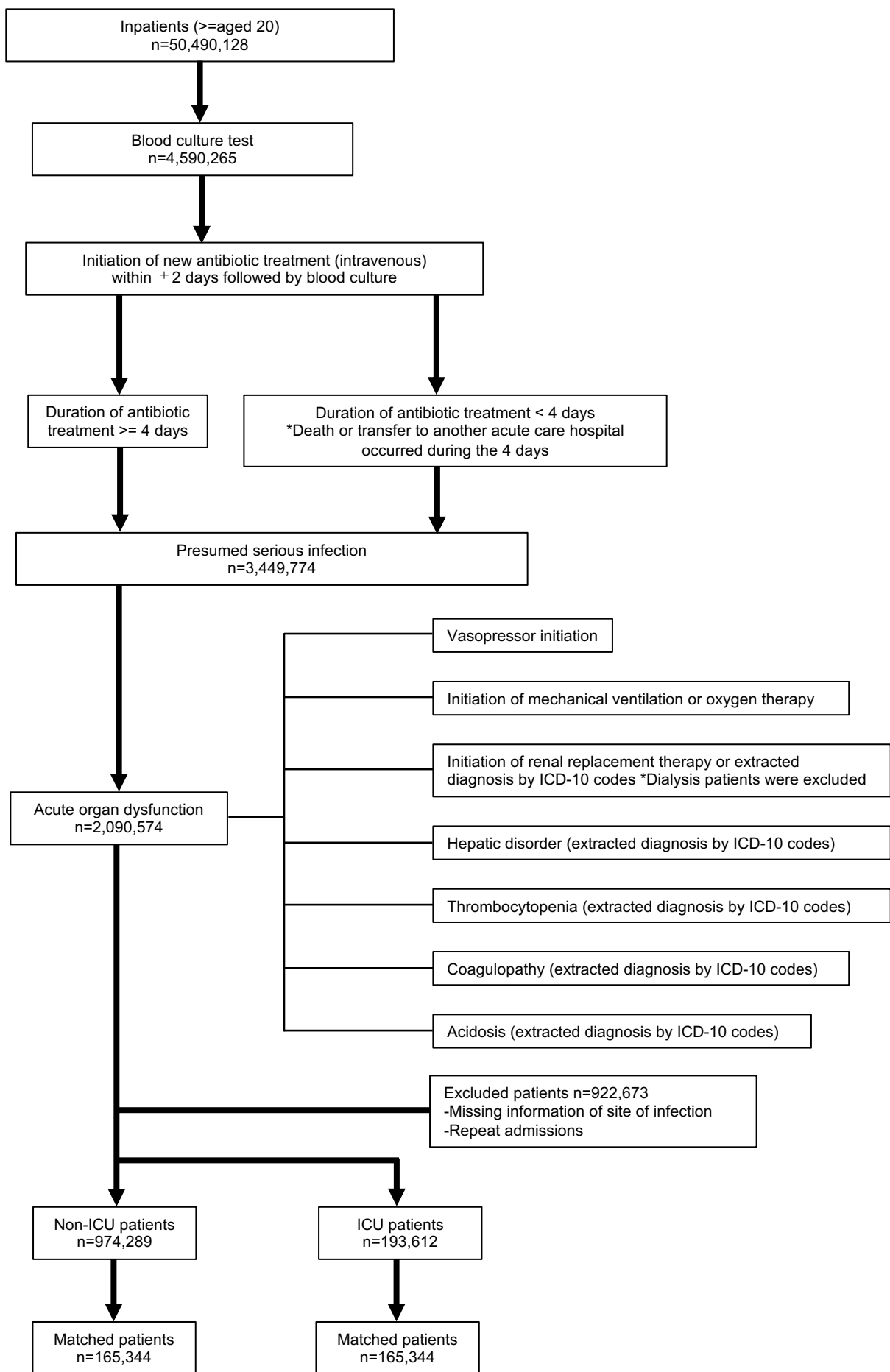

Supplement: Supplementary file 2 — Additional file 2: Figure S1. Flowchart of study population. [file 40560_2023_650_MOESM2_ESM.pdf]

A. Vasopressor

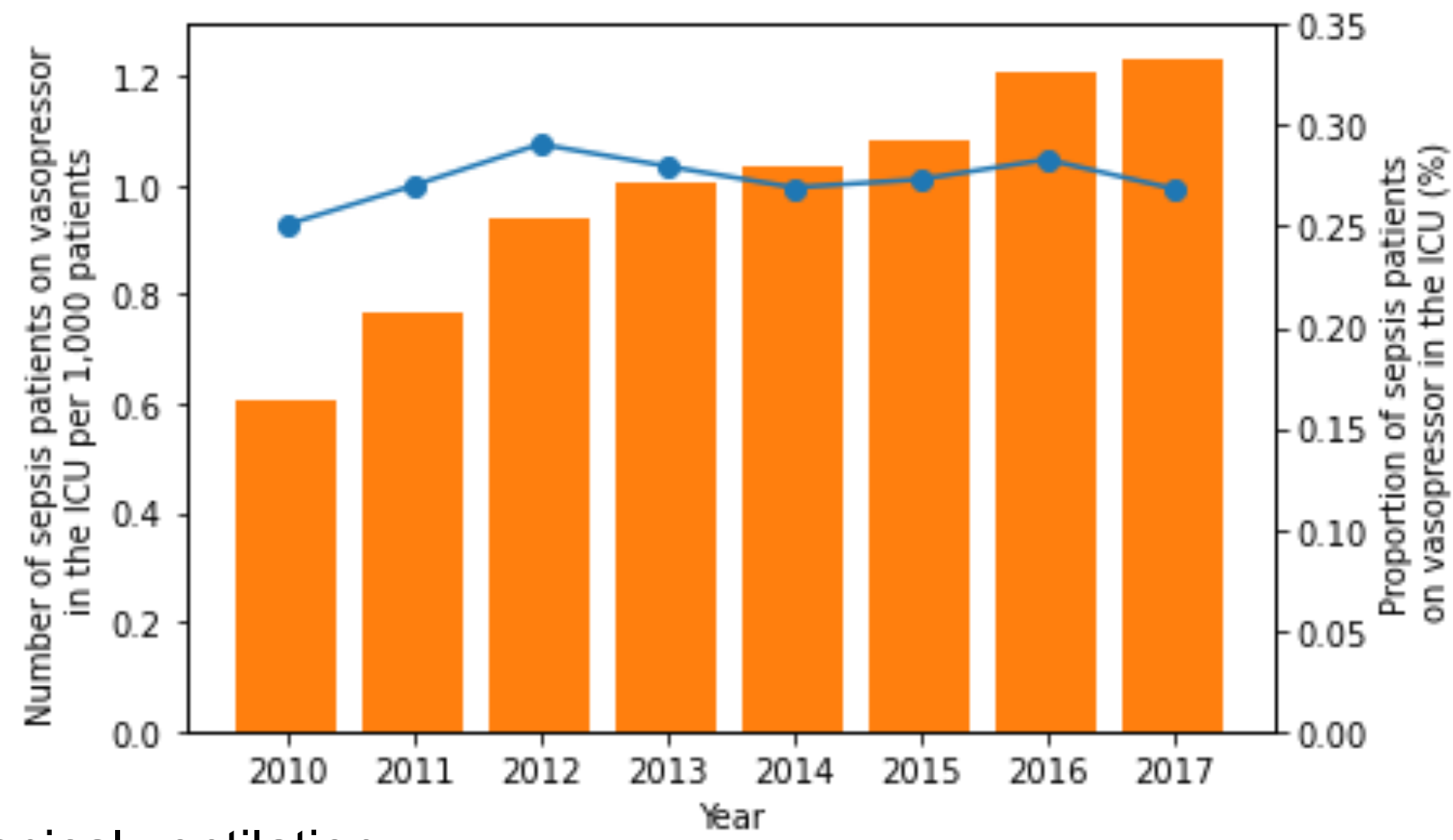

B. Mechanical ventilation

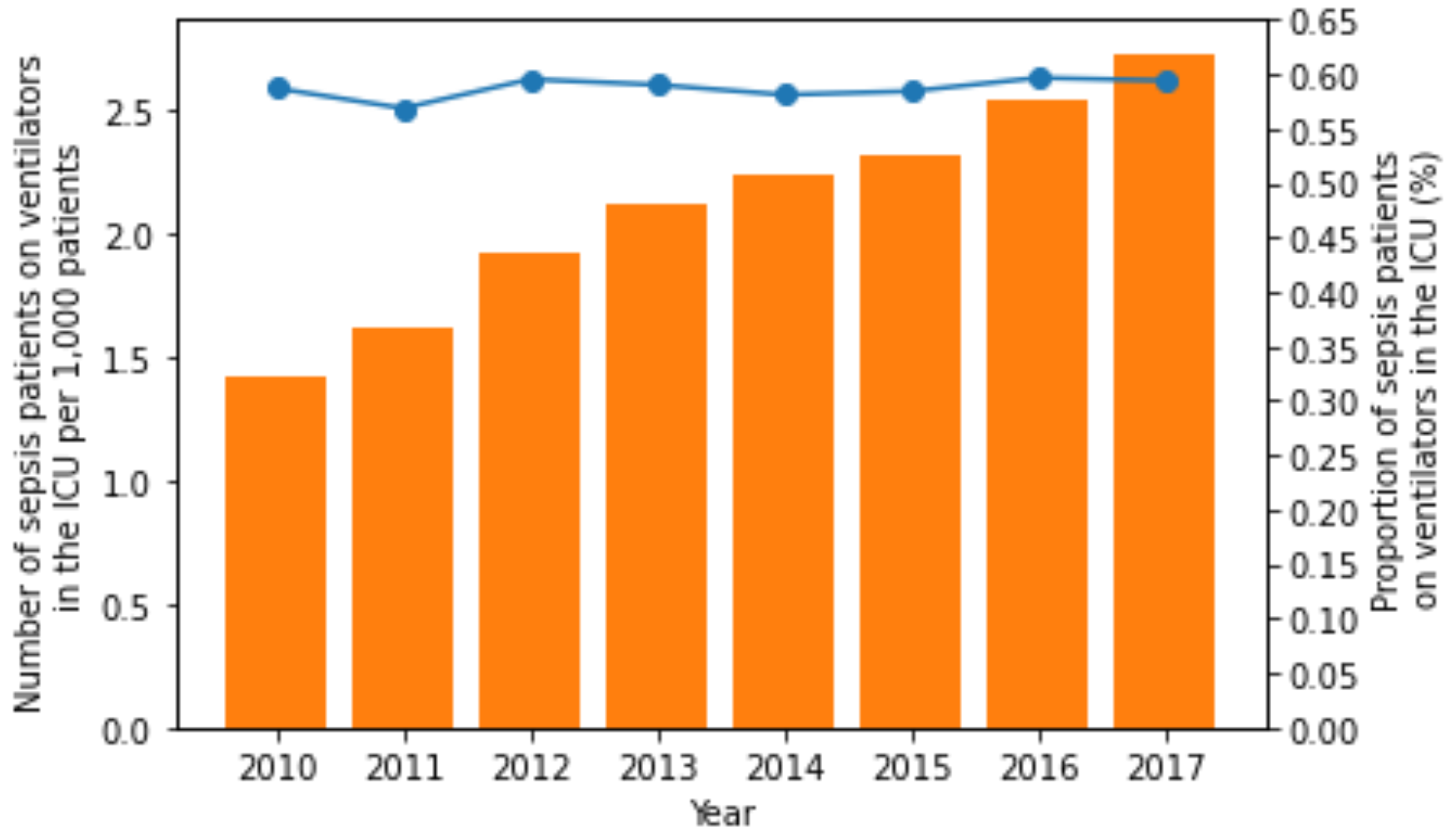

C. RRT

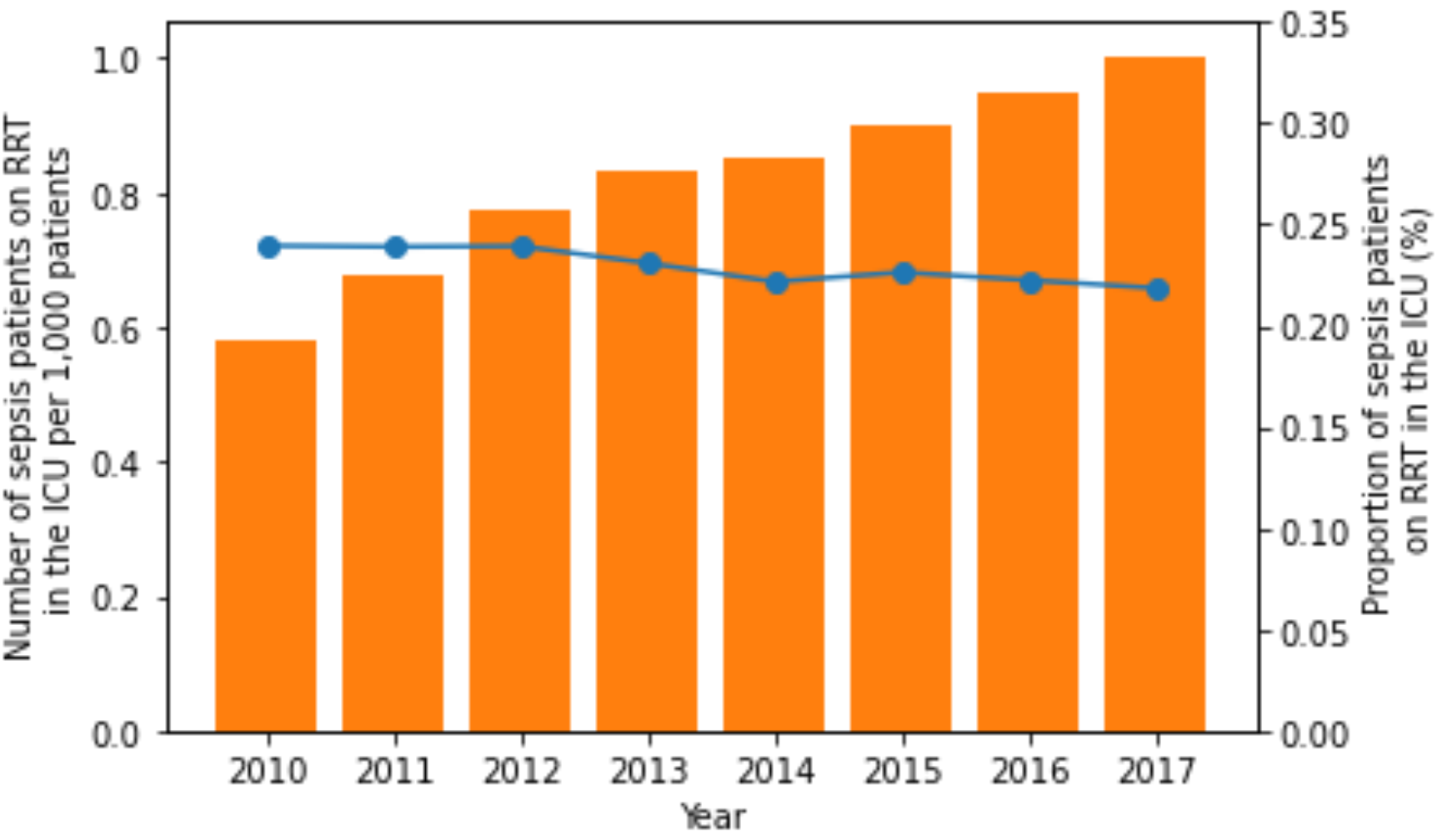

Supplement: Supplementary file 3 — Additional file 3: Figure S2. Temporal changes in the number and proportion of sepsis patients on therapeutic interventions in the ICU between 2010 and 2017. [file 40560_2023_650_MOESM3_ESM.pdf]

A

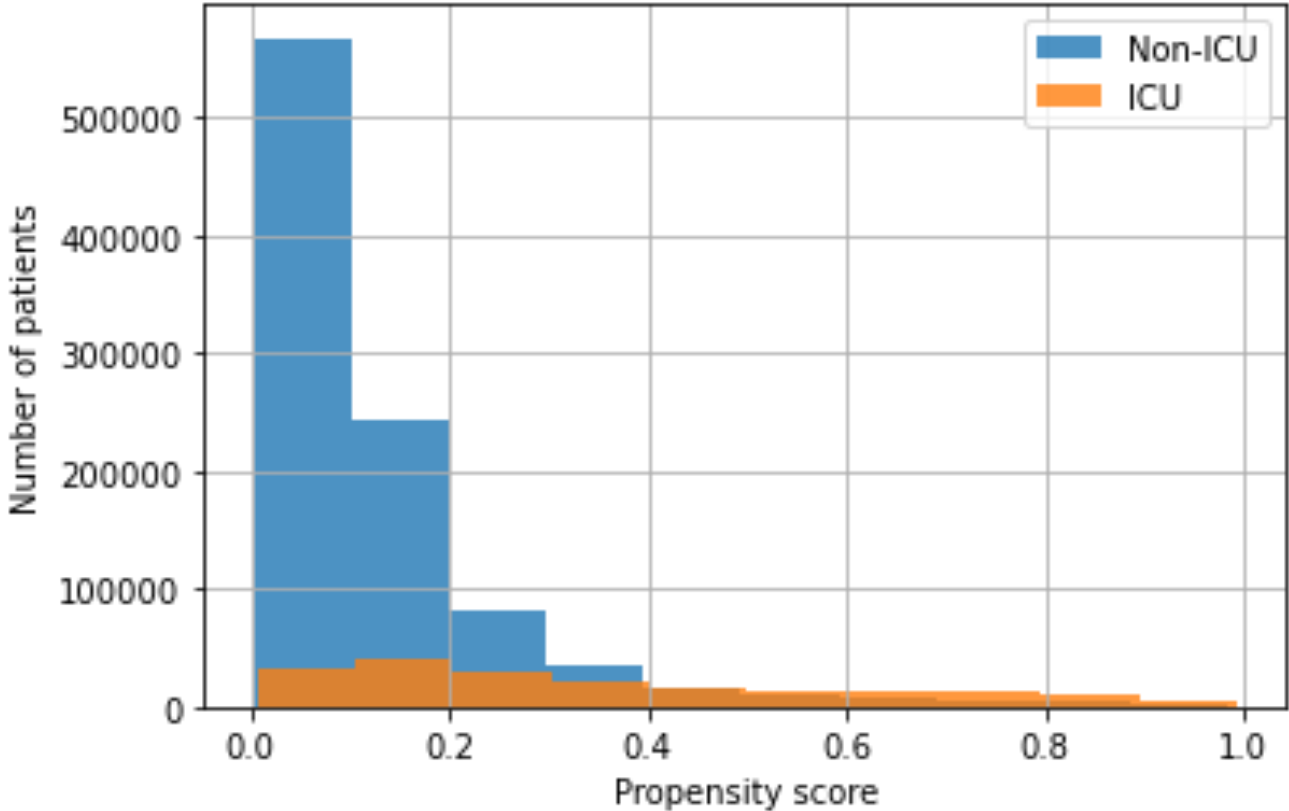

B

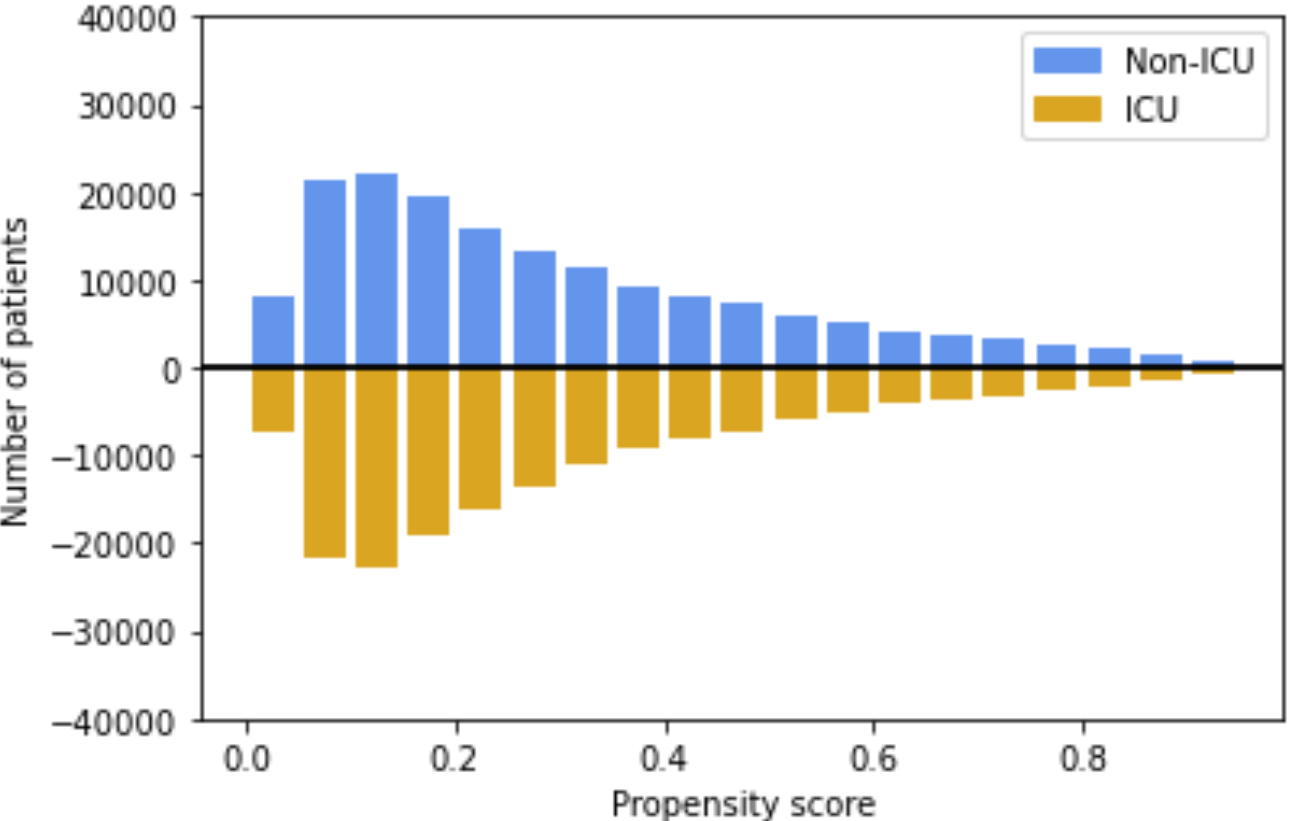

Supplement: Supplementary file 4 — Additional file 4: Figure S3. Distribution of propensity score matching. [file 40560_2023_650_MOESM4_ESM.pdf]
